# Reconstructive surgical therapy of peri-implant defects with ribose cross-linked collagen matrix and crosslinked hyaluronic acid – a prospective case series

**DOI:** 10.1007/s00784-024-05942-6

**Published:** 2024-09-20

**Authors:** Anton Friedmann, Rico Jung, Hakan Bilhan, Hanan Al Ghawi-Begovic, Frederic Kauffmann, Daniel Diehl

**Affiliations:** Department of Periodontology, Faculty of Health, Witten/Herdecke University, Alfred-Herrhausen-Str. 50, 58455 Witten, Germany

**Keywords:** Hypochlorite/aminoacid gel, Crosslinked hyaluronic acid, RCLC, Peri-implant defect, Decontamination, Reconstructive surgery, xHyA

## Abstract

**Objective:**

To investigate the efficacy of ribose-crosslinked collagen (RCLC) matrices functionalized by crosslinked hyaluronic acid (xHya) for reconstructive treatment of class I and III (b-c) peri-implantitis lesions in a transmucosal healing mode.

**Materials and methods:**

Thirteen patients presenting with 15 implants were included in this prospective case series. Upon flap reflection, the implants were thoroughly decontaminated employing glycine powder air polishing and adjunctive sodium hypochlorite. For defect augmentation, xHyA was administered to the bony defect walls, exposed implant surfaces, and the RCLC matrix before defect grafting. The full-thickness flap was readapted and sutured around the implant neck for transmucosal healing. Baseline and respective values at the 12 months post-op evaluation were recorded for the clinical parameters peri-implant probing depth (PPD), buccal soft tissue dehiscence (BSTD) and bleeding on probing (BoP). Furthermore, two independent investigators analyzed radiographic changes in the defect area. The mean changes for all variables were analyzed with a paired t-test.

**Results:**

The initial mean PPD was 7.2 ± 1.9 mm, and BoP was present in 63% of sites. After 12 months, PPD at the latest visit was 3.2 ± 0.66 mm, which amounted to a respective 3.9 ± 1.85 mm reduction, while the BoP frequency dropped to 10% at all sites. Radiographic bone fill was accomplished for 62.8% of the former defect area, accompanied by a mean MBL gain of 1.02 mm around the treated implants (all *p* < 0.001).

**Conclusions:**

Within the limits of this case series, we conclude that the proposed treatment sequence substantially improved peri-implant defects and offered a simplified but predictive technique.

**Clinical relevance:**

Reconstructive treatment approaches for peri-implantitis are effective but remain non-superior to open flap debridement. Further research on novel biomaterial combinations that may improve reconstructive treatment outcomes are warranted. Ribose-crosslinked collagen matrices biofunctionalized by hyaluronic acid used in this study yield improved clinical and radiographic peri-implant conditions after 12 months.

**Graphical Abstract:**

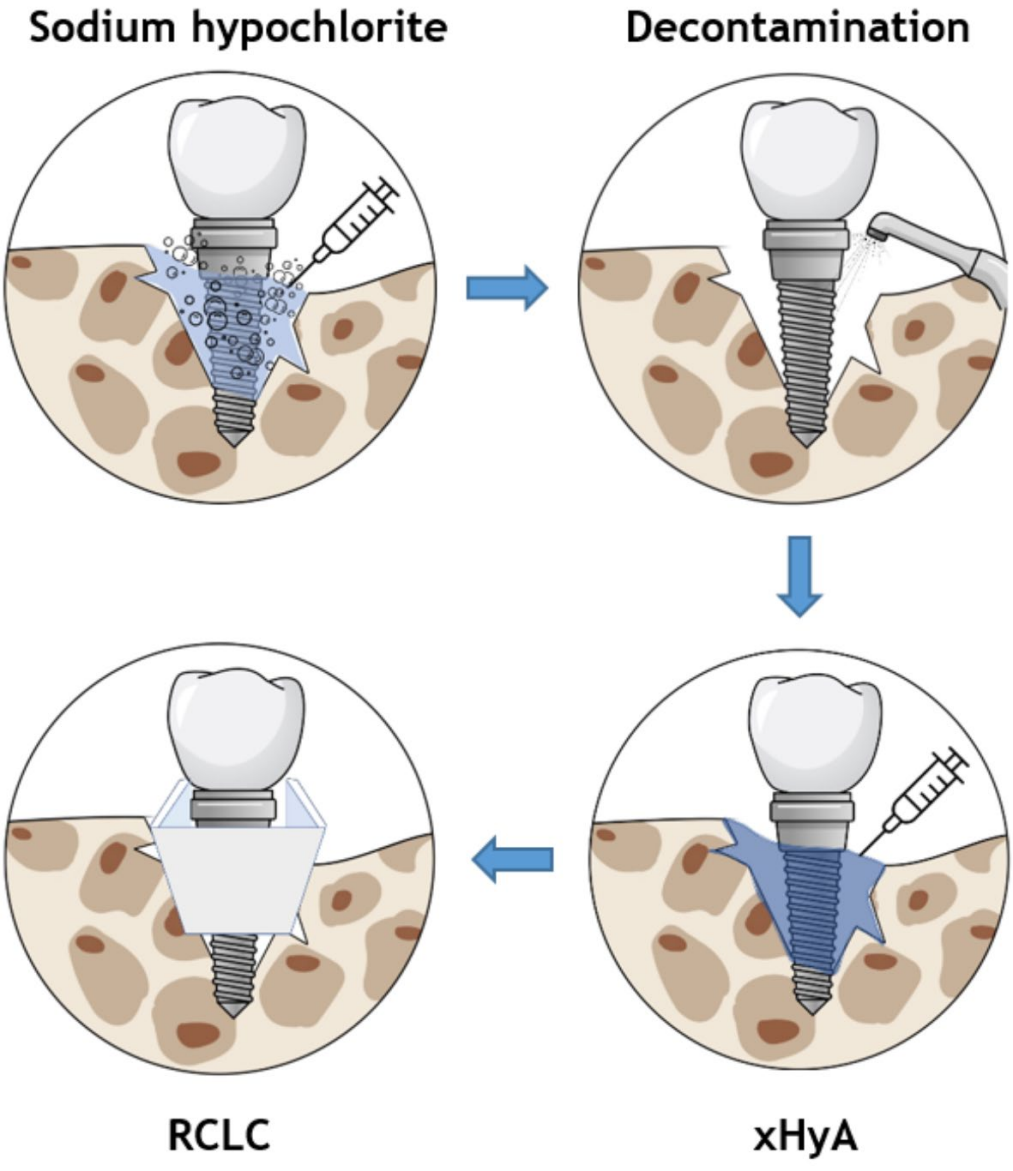

**Supplementary Information:**

The online version contains supplementary material available at 10.1007/s00784-024-05942-6.

## Introduction

Peri-implantitis is an infectious condition affecting the surrounding tissues of osseointegrated implants. The disease is characterized by inflammatory markers like bleeding or suppuration, increased probing depth, and radiologically verified loss of peri-implant bone [1]. Treating severe peri-implantitis focuses on resolving the inflammation and modifying the defect morphology to reduce the intrabony defect component [2]. However, non-surgical treatment has repeatedly been shown to have limited efficacy in managing most cases of peri-implantitis as opposed to periodontal therapy [3]. The surgical approach is, therefore, considered the most efficacious in resolving peri-implant inflammation and arresting bone loss when severe peri-implantitis is treated [4–7].

The primary aim of surgical therapy is to gain access to the implant threads and effectively remove calculus and plaque deposits. Apart from implant decontamination, the choice between a resective or reconstructive approach is generally influenced by the defect configuration since the peri-implant defect morphology substantially affects the outcome of regenerative healing [8–10].

The reconstructive therapy of implants with severe peri-implant defects has been extensively discussed in the literature. Numerous treatment protocols have been introduced and analyzed in the past decade, and many biomaterial combinations have been proposed [11–13]. The most common approach is guided bone regeneration (GBR), usually conducted with xenografts, autogenous bone, or allografts applied with a preferably resorbable membrane separating the grafted defect area from contacting the soft tissue flap.

According to the recent clinical practice guideline on the management of peri-implantitis, however, reconstructive procedures are as recommended as access flap surgery for peri-implant osseous defects. Moreover, various systematic reviews and meta-analyses suggest that a standard of care regimen for reconstructive peri-implant treatment can still not be drawn from the clinical literature, indicating that further research into reconstructive technologies to improve reconstructive therapies for peri-implantitis is still warranted [2, 14–16].

Consequently, this prospective case series aimed to validate the effectiveness of a novel reconstructive therapy approach utilizing a ribose cross-linked collagen matrix biofunctionalized with hyaluronic acid.

## Materials and methods

### Patient selection

This prospective case series enrolled 13 non-smoking patients (5 males, 8 females) aged 31–79 years, 10 of whom also had been diagnosed with stages 2 or 3 periodontitis (Table 1). These 13 patients presented with 15 implants exhibiting periimplantitis, as diagnosed by probing pocket depth ≥ 6 mm and radiologically verified peri-implant bone loss below the implant shoulder. All implants presented an intrabony defect morphology corresponding to classes I and III, b-c, according to Monje et al. [17]. Implants exhibiting other defect classes or advanced bone loss exceeding 75% were excluded. The Ethics Committee of Witten/Herdecke University approved the study protocol (S-174/2022), and all patients gave written informed consent for the procedure, follow-up visits, and the use of clinical data for research purposes.

**Table 1:** Patient and implant-related demographics

Mean Age (Years)		64.3 ± 9.75
Total n		13
Implants	Total	15
	Sites	79
Suprastructure	Crowns	9
	FPD	6
Gender	Female	43%
	Male	57%
Periodontal Diagnosis	Grade A	2
	Grade B	5
	Grade C	3
Radiographic bone loss	> 50%	6
	< 50%	9

### Presurgical treatment

All periodontitis patients received comprehensive systematic periodontal treatment, including oral hygiene instructions at peri-implantitis sites whenever necessary. Implant-supported dental crowns with limited cleanability were intentionally reshaped to ensure accessibility of interdental brushes and other hygiene measures. During systematic non-surgical therapy, all included implants were thoroughly cleaned using titanium curettes (Arnold Deppeler SA, Rolle, Switzerland). Non-periodontitis patients received equal non-surgical therapy at diseased implant sites.

### Surgical procedure

**Fig. 1:**
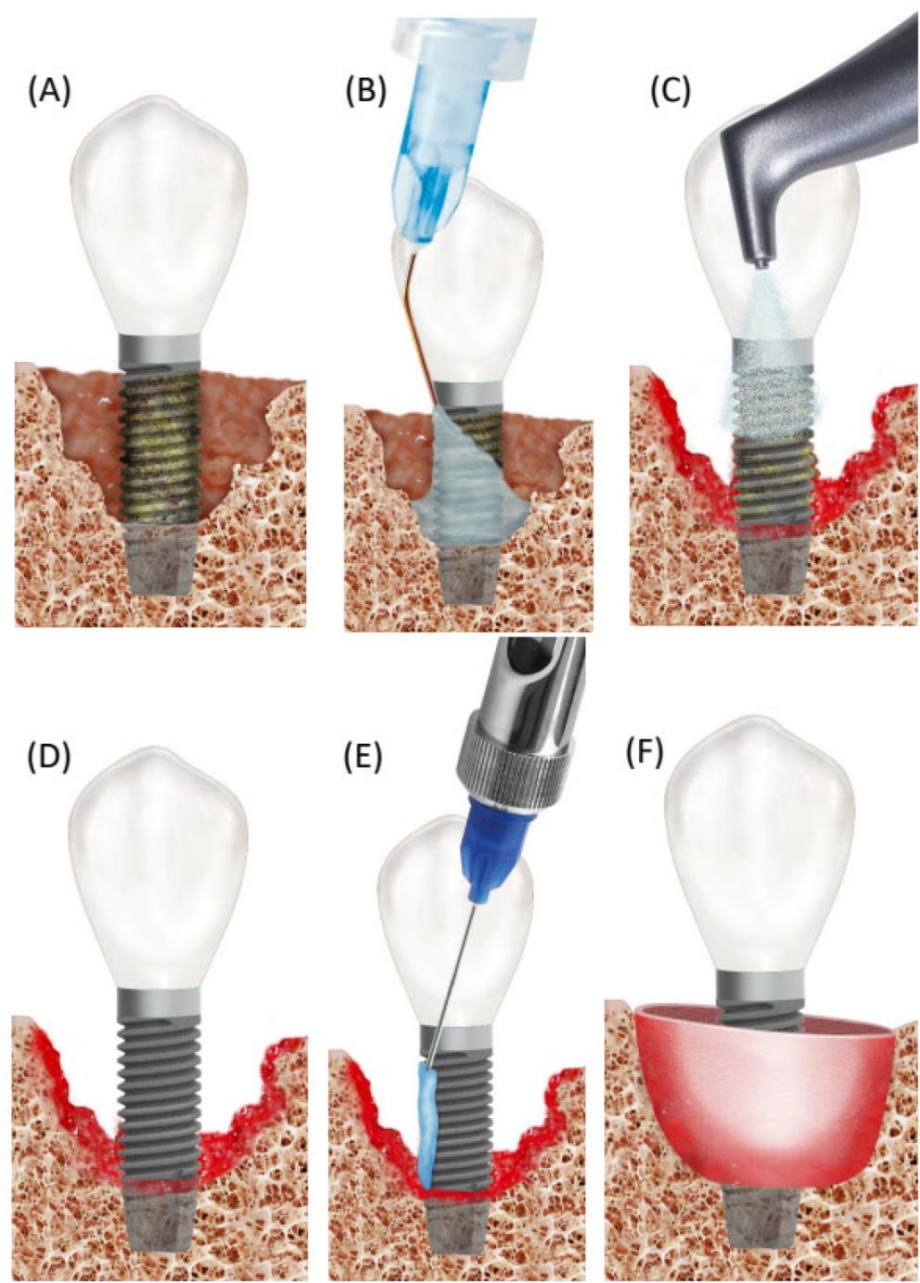
Schematic representation of the proposed workflow. After flap reflection, implants exhibiting intrabony defects (**A**) are thoroughly decontaminated using sodium hypochlorite gel (**B**) and glycine air powder abrasion (**C**). The decontaminated implant surfaces (**D**), as well as the defect, are coated with xHya (**E**). The glycation cross-linked collagen matrix provides both an osteoconductive scaffold and a barrier from the soft tissues (**F**)

**Fig. 2:**
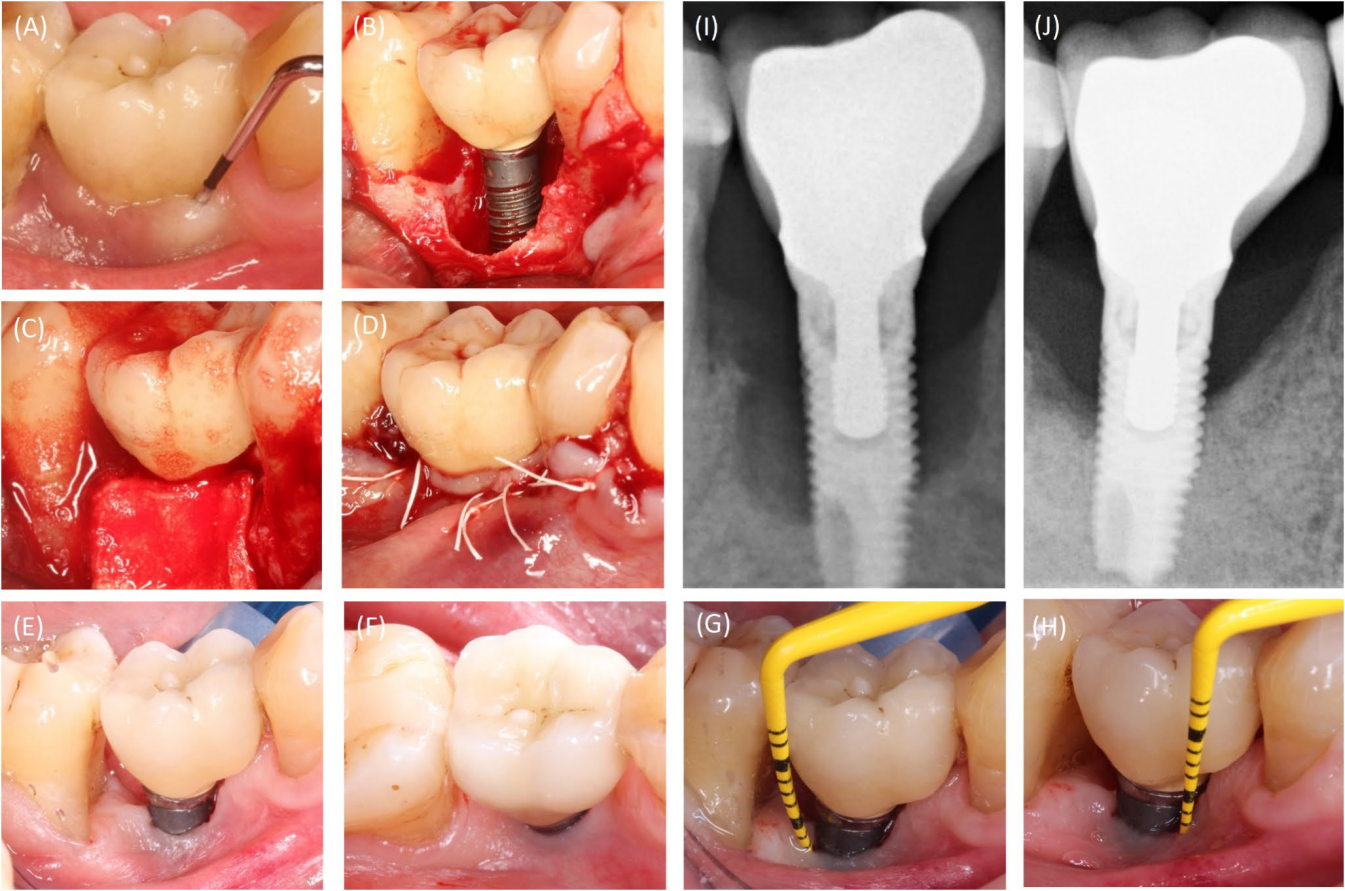
Case illustration for the proposed treatment protocol. (**A**) Implants presenting with PD > 8 mm and suppuration. (**B**) Full-thickness flap elevated using modified papilla preservation technique. (**C**) xHya-functionalized crosslinked collagen matrix applied to the defect. (**D**) Sutures in place. (**E**-**H**) One-year post-op, the implant exhibits no signs of inflammation or increased PPD. (**I**-**J**) X-Rays of the preoperative situation and one year postoperative

The included cases were treated by 4 calibrated and equally trained periodontists (A.F., H.B., R.J., D.D.). All surgeries were performed under local anesthesia (4% articain, 1:100000, Ultracain DS forte, Septodont, Germany) without removing the prosthetic frameworks. A marginal incision was made, and the implant-related papillae were preserved utilizing the modified papilla preservation technique (MPPT). No vertical releasing incisions were performed [18, 19]. A full-thickness flap was elevated, and granulation tissues were removed by sharp dissection (Fig. 1B). During defect debridement, adjunct sodium hypochlorite/amino acid gel (Perisolv, Regedent AG, Switzerland) was administered to the defect and implant surface for 30–45 s (Fig. 2B) to aid thorough surface decontamination. Mechanical calculus and plaque removal were achieved by glycine powder air polishing (Airflow, EMS, Basel) and titanium curettes. The decontamination sequence was repeated for a total of three times on each implant (Fig. 2B + C). After complete decontamination (Fig. 2D), both, the exposed implant surface and the defect walls were covered with 1.4-butanediol diglycidyl ether (BDDE)-crosslinked hyaluronic acid gel (xHyA, Hyadent BG, Regedent AG, Switzerland, Fig. 2E) until plenished. Then, a ribose cross-linked collagen matrix (RCLC, Ossix Volumax, Regedent AG, Switzerland) was soaked with xHyA, folded twice as indicated (Fig. 2F + 2C), and then adapted closely into the space between the implant surface and the residual intraosseous bone walls with a blunt instrument. The matrix was strictly limited to the intrabony defect components. In cases with buccal bone dehiscence, part of the matrix was placed overlapping the buccal bone margins to prevent the flap from collapsing into the defect (Fig. 1C). Flaps were closed tensionless employing coronally advanced flap technique at the emergence of the implant and sutured with modified mattress sutures and single stitch sutures using PTFE (5.0 Biotex, Purgo Biologics Inc., Challans, France) and resorbing (Monocryl 5.0, Ethicon, Hamburg, Germany) suture materials (Fig. 1D). Sutures were removed 14 days postoperative.

### Post-operative instructions

Patients were instructed to abstain from mechanical oral hygiene measures for 6 weeks and to apply chlorhexidine topically (Chlorhexamed, GlaxoSmithKline Consumer Healthcare GmbH & Co. KG, Munich, Germany (0.2%)) instead. Therefore, patients were instructed to switch to chlorhexidine gel (1%) after the suture removal for 4 consecutive weeks. Doxycycline 200 mg was administered in 1 daily dose for 10 days post-op, while analgesics (Ibuprofene 600 mg) were used upon individual need.

### Clinical parameters

Healing was monitored weekly during the initial 6 weeks post-operative; clinical follow-up, including assessment of plaque and bleeding scores, was scheduled periodically at supportive periodontal therapy (SPT) visits 12 weeks post-operative and quarterly thereafter. The clinical parameters peri-implant probing depth (PPD) change, buccal soft tissue dehiscence (BSTD), and bleeding on probing (BoP) were assessed by the operators 12 months after surgery. The parameters BoP and PPD were assessed at all diseased sites according to inclusion criteria. PPD was measured first, and the presence or absence of bleeding was noted for every probed site afterward. The radiographically indicated defect change was assessed by two separate calibrated investigators who weren’t involved in the clinical procedures (H.A.G.-B. and F.K.).

All assessed parameters except BoP were statistically analyzed with a paired t-test (α = 0.05) using Prism 9 software (Graphpad Software Inc., Boston, MA, USA).

### Radiographic follow-up

**Fig. 3:**
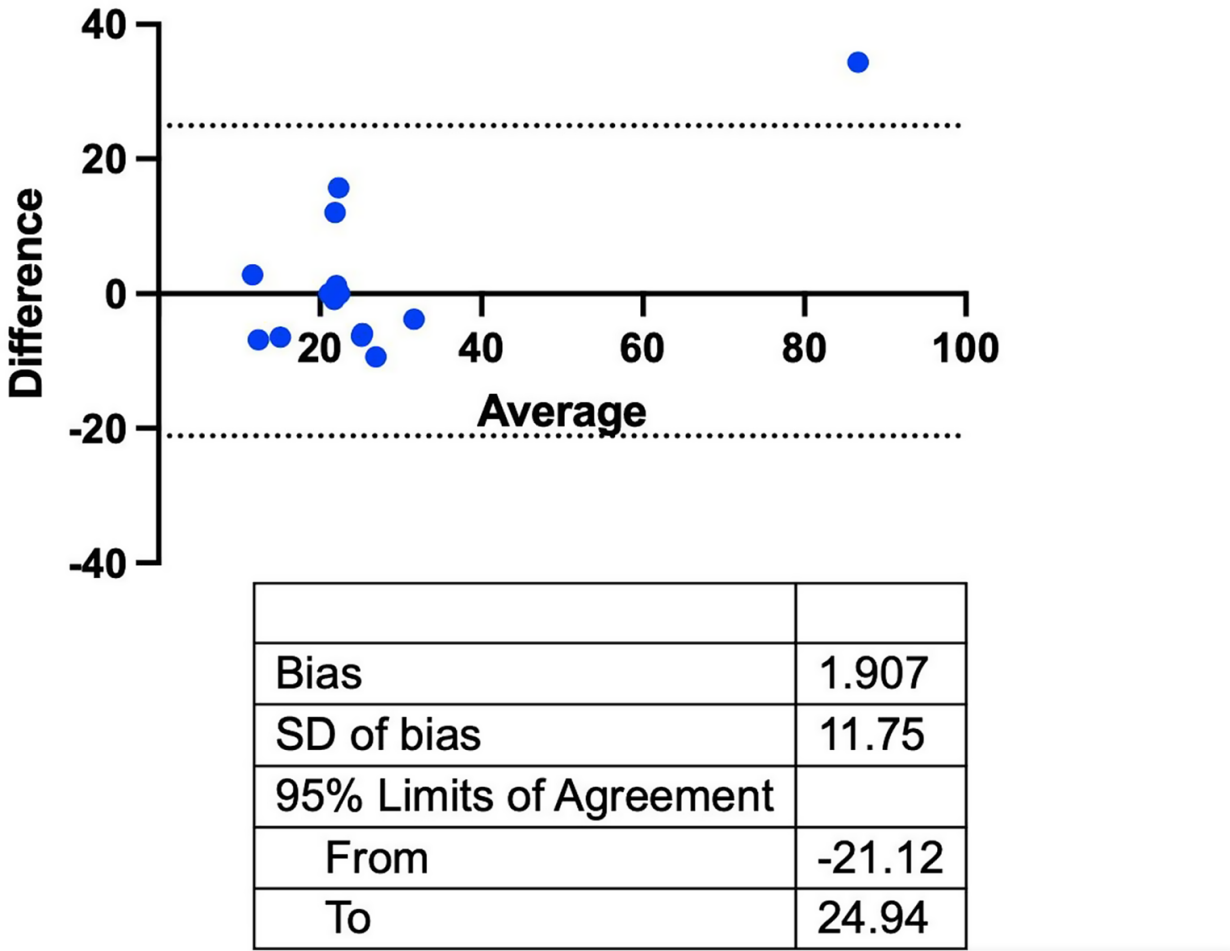
Bland-Altman plot for the interrater reliability of two investigators who analyzed radiographic images. Blue dots represent the deviation from the mean of both measurements. The dotted lines represent 95% limits of agreement

Baseline radiographs were taken during each patient’s treatment planning phase, within 4–6 weeks prior to the surgical procedure. Twelve months post-op, another X-ray was taken using a parallel technique, and both images were analyzed using ImageJ software (NIH, Bethesda, USA). The baseline and the latest periapical x-ray were corrected for the respective implant diameter. The total change in marginal bone level (MBL) and the percentage area gain were measured and calculated [20]. In brief, the lining tool from ImageJ was used to measure the distance from the radiographic implant shoulder to the deepest point of the intraosseous component (MBL). For the gain in radiographic defect fill, the defect-limiting bone walls were marked to measure the resulting surface areas. Two independent investigators performed all radiographic analyses. The intrarater reliability (R^2^ = 0.97; R^2^ = 0.94) was assessed by Pearson correlation coefficient of two independent measurements that took place one week apart (Supplementary Fig. 1). The interrater reliability for the measurements was assessed *post hoc* by Bland-Altman-Plot (Fig. 3), and the means and standard deviations from both investigators were used for further statistical analysis by paired t-test (α = 0.05). All analyses were performed with Prism 9 software (Graphpad Software Inc).

## Results

**Fig. 4:**
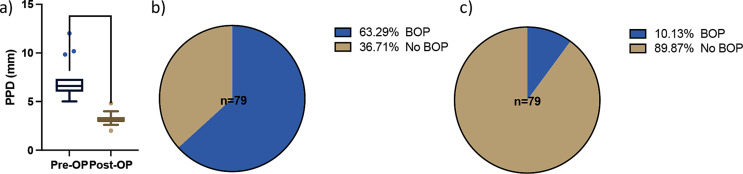
Mean PPD (**a**) and BOP (**b** + **c**) values. ****p < 0.0001

This case series included 13 patients and 15 implants (Table 1) with an initial mean probing depth of 7.2 ± 1.9 mm (Table 2; Fig. 4). BoP was present in 63% of sites before treatment (Table 2; Fig. 4). Two implants exhibited an insufficient amount of keratinized mucosa and were thusly treated with a free gingival graft 12 weeks before reconstructive surgery. All treated implants healed uneventfully, none of the surgical sites required premature intervention, and all sites presented with complete closure by week six after surgery. All implants were followed up for at least 12 months. Based on the latest examination in every case, the number of sites exhibiting BoP was substantially reduced to 8 out of 79 (10%) from 15 implants, which resulted in a statistically significant reduction of bleeding frequency (*p < 0.0001*).

**Table 2:** Clinical and radiographic data from baseline to final evaluation

		Pre-op	Post-op	∆	*p*
Clinical data	**Mean PPD** (mm ± SD)	**7.2 ± 1.9**	**3.2 ± 0.67**	**3.94 ± 1.85**	**< 0.0001**
	**Mean BSTD** (mm ± SD)	**0.56 ± 1.22**	**2.43 ± 0.93**	**1.87 ± 0.40**	**< 0.0001**
	**Mean BoP (%)** **n BoP / n total**	**63%** **46 / 79**	**10%** **7 / 79**	**n.a.**	**< 0.0001**
Radiographic data	**MBL** (mm ± SD)	**5.83 ± 2.63**	**4.81 ± 2.57**	**1.02 ± 0.64**	**< 0.0001**
	**Defect area** (mm^2^ ± SD)	**45.94 ± 26.91**	**18.29 ± 12.21**	**27.65 ± 18.50**	**< 0.0001**

The probing depth assessed at 12 months was 3.2 ± 0.66 mm, resulting in a probing depth reduction (ΔPPD) of 3.9 ± 1.85 mm (Fig. 4), accompanied by a slight BSTD of 1.87 mm ± 0.49. This reduction was statistically significant (*p < 0.0001*).

**Fig. 5:**
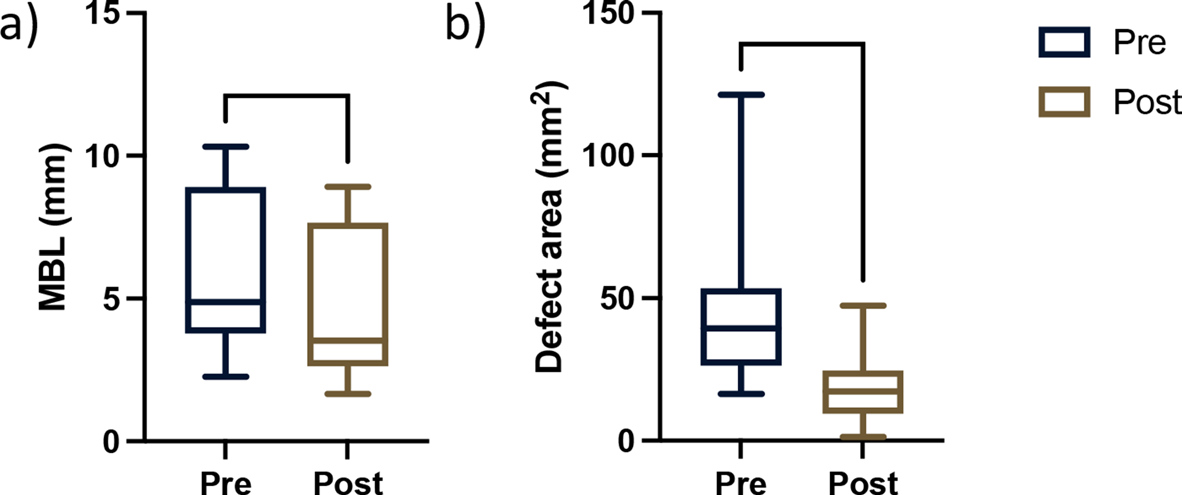
Mean marginal bone level (**a**) and defect area fill (**b**) by periapical x-ray analysis. ****p < 0.0001

Correspondingly, the defect extension exhibited significant reduction with a newly mineralized area of 27.65 ± 18.50mm^2^*(p < 0.0001*, IRR bias 1.907±11.75, Fig. 3*)*, which equaled 69.1% of mineralized tissue gain, as a result from pairwise X-ray analysis. Moreover, a significant MBL gain calculated from mesial and distal aspects of the 15 analyzed implants was found at an average of 1.01 ± 0.65 (Fig. 5).

## Discussion

The goal of peri-implantitis treatment is to resolve the inflammation to arrest progressive bone loss. Surgical procedures are generally more successful in achieving this aim. However, the evidence for an additional benefit of reconstructive approaches is still limited, although they represent a rational treatment option per se [21].

This case series demonstrates that the proposed reconstructive protocol combining xHyA with an RCLC matrix successfully improves peri-implant conditions expressed by clinical and radiographic parameters. The clinical parameters revealed healthy and non-inflamed peri-implant tissue conditions that remained stable to the final evaluation in almost all treated sites (Fig. 4 + 5). The radiographically indicated resolution of the intrabony defect component according to x-ray analysis was consistent in all treated implants.

In this study, the mean MBL gain was approximately 1.02±0.6 mm, accompanied by a significant defect area fill of 60% and a PPD reduction of 3.9±1.8 mm with a consecutive BSTD increase of 1.87 mm. According to more recent meta-analyses, the mean radiographic MBL gain after reconstructive peri-implantitis treatment ranges between 1.01 and 1.66 mm (CI = 1.23;2.09), accompanied by a mean PD reduction of 1.27 mm (CI = 0.6;-1.96) [22, 23] indicating that the proposed protocol yielded results expectable from the literature. Moreover, the results align with a recent multicentric randomized-controlled trial that monitored the reconstructive outcome for 12 months. Therein, Derks et al. reported a PPD reduction of 3.7 mm and an MBL gain of 1.0 mm [24]. However, a direct comparison to other reconstructive procedures is not entirely feasible. Even though, the choice between submerged and transmucosal healing cannot be made based on clinical evidence, the current guideline suggests a disconnection of the framework to allow a submerged healing mode [2]. The proposed concept, however, allows semi-open healing without removing the prosthetic framework. To our knowledge, this is one of few studies about reconstructive peri-implantitis treatment without submerged healing. A three-year observational case series reported successful reconstructive treatment of infrabony peri-implant defects. The reported outcome in 16 patients showed considerable improvement in clinical parameters and radiographic defect closure [12]. Moreover, in a randomized controlled trial treating peri-implant defects with bone substitutes and a collagen membrane in a transmucosal healing mode, Roos-Jansåker et al. reported a stable defect fill of 1.6 mm over three years [25]. Within this frame of reference, it appears reasonable to suggest that submerged healing may not be necessary in every case scenario, provided adequate wound closure can be achieved.

A recent meta-analysis revealed a mean soft tissue recession of approximately 0.4 mm for reconstructive peri-implantitis treatments [23]. With a BSTD of 1.87 mm, the implants treated in this study exhibited higher BSTD than expectable from the clinical literature. However, with a mean radiographic bone loss of 5.83 mm, all implants exhibited advanced bone loss to begin with. Considering the high heterogeneity in biomaterials and preoperative defects across all studies and the fact that the peri-implant attachment level gain is comparable to the literature, the relatively high BSTD may be related to the advanced preoperative defect depth.

Unfortunately, the literature on the reconstructive treatment of peri-implant defects exhibits high heterogeneity regarding the choice of biomaterials, with most studies proposing combinations of various bone substitutes and barriers. More recently, studies investigating the adjunctive reconstructive effect of biologics, such as enamel matrix derivatives, have reported promising results for reconstructing peri-implant defects [26, 27]. Until now, however, hyaluronic acid has not been investigated for this indication, even though clinical and experimental studies have confirmed the pronounced effect of xHyA on bone formation [28, 29]. For instance, the histomorphometric amount of newly organized bone was more pronounced in extraction sockets that received xHyA and a deproteinized bovine bone mineral (DBBM) composition than in those grafted by DBBM alone [30]. Similar results have been reported by Kauffmann et al. [31], who showed that xHyA increased bone volume after lateral guided bone regeneration (GBR).

For the adequate blood clot stability that is required for the regeneration of implant-supporting bone, most studies reported the use of a xenogenic or allogenic bone substitute [2]. However, the bone substitute rarely integrates into a functional hard-tissue matrix. Instead, the bone substitute material will be resorbed and replaced by newly formed bone over time, depending on the resorption kinetics of the respective material [32]. Conversely, collagen makes up 90% of the organic bone matrix, rendering it an ideal bone substitute in theory. However, native collagen underlies quick resorption, which leads to compromised graft stability [33, 34]. Therefore, RCLC membranes and matrices have been developed to increase durability and improve resorption kinetics [35]. Interestingly, these biomaterials have been shown to develop bone deposits over time, indicating that RCLC promotes actual bone matrix mineralization, rather than providing a scaffold for new bone matrix to form [36–38].

To the best of our knowledge, this is the first report of reconstructive peri-implantitis treatment using an RCLC matrix to augment the intrabony defect instead of particulate material, so clinical evidence is still scarce. However, considering the preclinical findings and the noticeable amount of newly formed bone in this report, slowly resorbing collagen matrices appear to be a reasonable material that warrants further investigation. Moreover, this effect may have been amplified by the combination with xHyA, as recent findings suggest that the latter reduces the resorption rate of collagen in diabetic rats [39, 40]. However, the specific contribution of xHyA and RCLC to the radiographic outcome presented here needs to be further elucidated. Moreover, the efficacy of both biomaterials remains to be investigated in a submerged healing mode.

The efficacy of implant decontamination is a major goal of peri-implantitis treatment and has, therefore, been the subject of several pre- and clinical studies. The clinical literature indicates that the modern semi-rough implant surface will likely become sufficiently decontaminated by various methods. At the same time, the degree of invasiveness oscillates among the applied chemical, mechanical, or energy-based treatments [41]. The hypochlorite/aminoacid gel used in this series was shown to significantly increase pocket depth reduction in a randomized controlled trial of non-surgical peri-implantitis. Likewise, several in vitro studies pointed out the strong antimicrobial effect of sodium hypochlorite cleaning gel [42, 43]. Considering that decontaminated implant surfaces are a prerequisite for successful regeneration and treatment, the adjunctive use of sodium hypochlorite gel appears reasonable and proved to be effective, as expressed by the clinical results presented. However, a measurable adjunctive effect of the cleaning gel in conjunction with the more common glycine powder air-polishing remains a matter of speculation at this point.

As a prospective case series, this study exhibits some considerable limitations, such as the absence of a control group. However, it is evident from recent meta-analyses and clinical treatment guidelines that open-flap debridement and resective approaches are successful in terms of resolving inflammation. It is, therefore, not obvious that a control group treated with a different approach would have improved the quality of this proof-of-concept study. Nonetheless, it would be of interest to include more treatment arms in the future to further evaluate the adjunctive effects of xHya and sodium hypochlorite cleaning gel in comparison to more established bone substitutes. Furthermore, no standardized patient-reported outcome measures (PROM) were assessed in this study. However, since healing was uneventful in all patients, we hypothesize that PROM is generally positive in response to this treatment. Nevertheless, this must be experimentally verified, especially in comparison with further study arms.

Taken together, our results suggest that a treatment protocol encompassing adequate implant decontamination by mechanical and chemical means and the reconstruction with RCLC biomaterials and xHyA is a viable approach for reconstructive peri-implantitis treatment. Furthermore, no prosthesis had to be removed, and the initially presented reconstructions stayed in function throughout the 12-month observation period, which indicates that submerged healing may not always be necessary. Nevertheless, more adequately controlled and prospective studies with larger cohorts are warranted to elucidate the factors contributing to positive treatment outcomes.

## Conclusions

Within the limits of a case series, we conclude that the proposed approach contributes valuable improvement to peri-implant conditions in previously affected implants and has a sustainable chance of maintaining these implants and reconstructions under improved biological conditions at a small risk of disease recurrence.

## Electronic supplementary material

Below is the link to the electronic supplementary material.

Supplementary Material 1

## Data Availability

No datasets were generated or analysed during the current study.
