# Supplementary material for: Reconstructive surgical therapy of peri-implant defects with ribose cross-linked collagen matrix and crosslinked hyaluronic acid – a prospective case series
Source: Clin Oral Investig. 2024 Sep 20;28(10):536. doi: 10.1007/s00784-024-05942-6 (PMC11415415; doi:10.1007/s00784-024-05942-6)
Supplement: Supplementary file 1 — Supplementary Material 1 [file 784_2024_5942_MOESM1_ESM.pptx]

## Slide 1
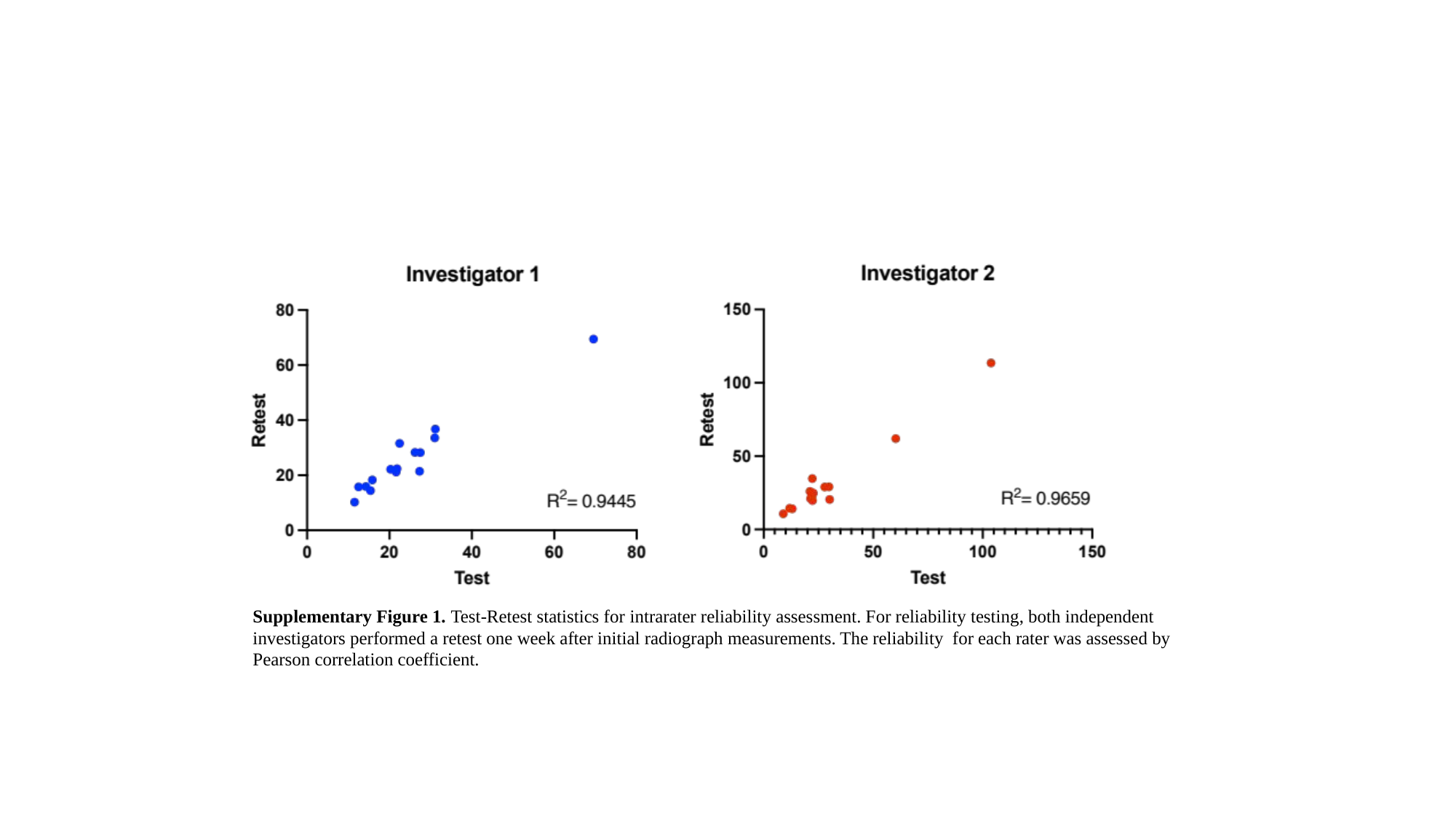

Supplementary Figure 1. Test-Retest statistics for intrarater reliability assessment. For reliability testing, both independent investigators performed a retest one week after initial radiograph measurements. The reliability for each rater was assessed by Pearson correlation coefficient.
